# Calorie Restriction with a High-Fat Diet Effectively Attenuated Inflammatory Response and Oxidative Stress-Related Markers in Obese Tissues of the High Diet Fed Rats

**DOI:** 10.1155/2012/984643

**Published:** 2012-06-19

**Authors:** Seungae Park, Na-Young Park, Giuseppe Valacchi, Yunsook Lim

**Affiliations:** ^1^Department of Food and Nutrition, Kyung Hee University, no. 1 Hoegi-dong, Dongdaemun-gu, Seoul 130-701, Republic of Korea; ^2^Department of Evolutionary Biology, University of Ferrara, 44100 Ferrara, Italy

## Abstract

Obesity characterized by increased mass of adipose tissue leads to systemic inflammation. Calorie restriction (CR) improves parameters associated with immune response and antioxidant defense. We hypothesized that CR with a high fat diet (HFCR) regulates local and systemic inflammation and oxidative stress damage in a high fat diet induced obesity (HF group). We investigated effect of HFCR on inflammation and oxidative stress-related markers in liver and adipose tissues as well as adipokines in plasma. HFCR lowered liver triglyceride levels, total cholesterol levels, and the plasma leptin/adiponectin ratio to normal levels and improved glucose tolerance. HFCR also improved fatty liver and normalized adipocyte size and morphology. HFCR reduced lipid peroxidation and decreased the expression levels of inducible nitric oxide synthetase, cyclooxygenase-2, NF-E2-related factor, and heme oxygenase-1 in the liver. Moreover, HFCR suppressed the expression levels of C- reactive protein and manganese superoxide dismutase in the adipose tissue in the HF group. These results suggest that HFCR may have beneficial effects on inflammation and oxidative stress as well as lipid profiles in the HF diet induced obesity. Moreover, HFCR may be a good way to increase compliance in obese patients and to prevent obesity induced complications without changes in dietary pattern.

## 1. Introduction

Obesity is a multifactorial disease resulting from a combination and interaction of genetic, environmental, psychological, social, and cultural factors [1, 2]. Obesity is considered a major public health problem because it is associated with insulin resistance, diabetes, hypertension, dyslipidemia, and coronary heart diseases and characterized by increased mass of adipose tissue, which is an active endocrine and secretary organ [3–5]. Adipocytes secrete a wide range of protein signals and factors including interleukin (IL)-6, IL-1*β*, tumor necrosis factor (TNF)-*α*, monocyte chemoattractant protein (MCP)-1, and adipokines such as adiponectin, leptin, and resistin [4, 5]. Therefore, obesity is a heightened state of inflammation [6]. An inflammatory process is characterized by increased expression of inflammatory markers and activated inflammatory signaling pathways such as Jun N-terminal kinase (JNK), I*κ*B kinase (IKK)-*β*, nuclear factor (NF)-*κ*B, and redox-sensitive transcription factor [4, 7]. Furthermore, body mass index and fat accumulation are positively correlated with levels of oxidative stress in human and animal models [8, 9]. Elevated oxidative stress induces insulin resistance by impairing phosphorylation of insulin receptor substrate (IRS)-1 and IRS-1-induced phosphatidylinositol 3 (PI3)-kinase activation, insulin-induced glucose uptake, and translocation of glucose transporter (GLUT)-4 [10].

A calorie restriction diet (CR), which is the reduction in calorie intake without malnutrition, improves many parameters involved in immune responses and antioxidant enzyme activities [11, 12]. Weight loss caused by moderate CR can lead to improving insulin sensitivity as well as reducing circulating inflammation-related products and increasing potent antiinflammatory factors produced by adipocytes [13–16]. CR and weight loss reduce the serum concentrations of IL-6 and C-reactive protein (CRP) in obese subjects and suppresses the upregulation of NF-*κ*B, cyclooxygenase (COX)-2, and inducible nitric oxide synthase (iNOS) in kidney [17–19].

Several studies have demonstrated that CR reduced the production of inflammatory cytokines such as TNF-*α* and IL-6 in healthy obese subjects [16, 20]. Although previous studies have examined the anti-obesity and antiinflammatory effect of CR on serum, liver, heart, and hypothalamus [21–25], there is only one previous study focused on the anti-obesity and antiinflammatory effect of CR in adipose tissue [21]. Moreover, the effect of CR on the expression of inflammatory markers such as iNOS, CRP, NF-E2-related factor (Nrf2), and heme oxygenase (HO)-1 in *in vivo* obese models continuously fed with a high-fat (HF) diet, is poorly documented.

We hypothesized that obese animals previously fed with the HF diet, when placed on HFCR, would see a reduction in inflammation and oxidative stress damage in obese tissues including adipose tissues.

## 2. Materials and Methods

### 2.1. Animals and Diets

Male Sprague-Dawley (SD) rats were obtained at 8 weeks old (Daehan Biolink Co., Eumseong, Chungcheongbuk-do, South Korea) and were individually housed in a temperature-controlled (22 ± 1°C) room on a 12 : 12 light-dark cycle and allowed free access to diets and tap water. After a 2-week acclimation period, the animals were randomly divided into two groups: a control diet group (CON, *n* = 20) and a high-fat diet group (HF, *n* = 40), after being balanced for body weights. Following 11–13 weeks of *ad libitum* access to a control diet (D12450B, 10% kcal fat; Research Diets, New Brunswick, NJ, USA) or a high-fat diet (D12451, 45% kcal fat; Research Diets), the CON was continuously provided with the control diet. The HF group was divided into two: (i) the high-fat diet group (HF, *n* = 20) and (ii) the high-fat diet group with calorie restriction (HFCR, *n* = 20) are fed their respective diets for 8–10 weeks. The HFCR group was fed 60% of the food intake from the previous day's amount of the HF group. During the experiment period, body weights were recorded weekly. After 8–10 weeks of treatment, the animals were fasted overnight, weighed, and anesthetized under ether. Blood samples were collected via cardiac puncture, and plasma was separated by centrifugation at 3000 rpm. Livers and fat pads including epididymal white adipose tissue (WAT) and retroperitoneal WAT were dissected and weighed. The tissues were isolated, frozen in liquid nitrogen, and stored at −80°C until analysis. All rats were used in accordance with animal protocols approved by the Kyung Hee University Institutional Animal Care and Use Committee.

### 2.2. Intraperitoneal Glucose Tolerance Test (IPGTT)

Glucose tolerance tests were carried out after 8–10 weeks of calorie restriction treatment. After an overnight fast, the rats were intraperitoneally (i.p.) injected with 50% glucose (2 g/kg body weight). Blood samples were collected from the tail at 0, 15, 30, 60, 90, and 120 minutes for glucose level measurements. The integrated area under the glucose curve (AUC) in the IPGTT was calculated by the trapezoid method from the glucose measurements at 0, 15, 30, 60, 90, and 120 min (mg/dL X min).

### 2.3. Histological Analysis

Histological sections (4 *μ*m thickness) were prepared from liver and epididymal WAT fixed in 10% buffered formaldehyde and embedded in paraffin. Histological sections were stained with hematoxylin and eosin (H and E).

### 2.4. Measurement of Lipid Peroxidation

Malondialdehyde-thiobarbituric acid (MDA-TBA) formation was used as an index of lipid peroxidation [26]. Briefly, 200 *μ*L of 8.1% SDS, 3 mL of 20% acetic acid-0.8% TBA mixture, and 600 *μ*L of distilled water were added to 0.2 mL of homogenated liver tissues with 0.15 M KCl buffer (10%, w/v), and heated for 60 min at 95°C. After cooling in ice, 1 mL distilled water, 5 mL mixture of n-butanol, and pyridine (15 : 1, v/v) were added and centrifuged at 4,000 rpm for 10 minutes. The upper layer was aspirated out, and fluorescence was measured with an ELISA reader at 532 nm as compared with 1,1,3,3-tetramethoxypropane.

### 2.5. Measurement of Lipid Content in Liver

Hepatic lipids were extracted using the Bligh and Dyer method [27]. Briefly, 1.25 g of tissues was homogenized with 3.75 mL of chloroform-methanol (1 : 2, v/v) using a homogenizer. After vigorous vortexing for 15 minutes, the homogenate was mixed with 1.25 mL of chloroform and an equal volume of water, then centrifuged briefly at 3000 rpm for 10 minutes. The lower phase was transferred into a new tube, and the residue was mixed with 1.88 mL of chloroform for the second-step vortex and centrifugation. The lower phase obtained by the centrifugation was mixed with the first chloroform phase in the same tube. After evaporation under nitrogen gas at 55°C, the lipid extract was dissolved in 2 mL of 2-propanol. Total cholesterol and triglyceride contents were analyzed by enzymatic methods (Bio Clinical System Co., Anyang, Gyeonggi-do, South Korea).

### 2.6. Measurement of Plasma Adipokine Concentrations

Leptin (R&D Systems, Minneapolis, MN, USA) and adiponectin (Adipogen, Seoul, South Korea) concentrations were determined by means of commercial radioimmunoassay kits according to the manufacturer's manual.

### 2.7. Western Blot Analysis in Liver and Epididymal WAT

The tissue was homogenized in lysis buffer and centrifuged at 14,000 rpm (30 min, 4°C). For Western blot analysis, equal amounts of protein (40 *μ*g per lane) were loaded in the wells of 8–10% polyacrylamide gels. After the electrophoretic run, proteins were electroblotted on polyvinylidene fluoride (PVDF) membranes (Millipore, Marlborough, MA, USA). The membrane was blocked by incubation in 5% non-fat milk in PBS-Tween 20, incubated with polyclonal antibody against p65 (Cell Signaling, Danvers, MA, USA, 1 : 100), TNF-*α* (Santa Cruz Biotechnology, CA, USA, 1 : 100), pI*κ*B*α* (Santa Cruz Biotechnology, 1 : 100), iNOS (Santa Cruz Biotechnology, 1 : 1000), CRP (Abcam, 1 : 200), COX-2 (Santa Cruz Biotechnology, 1 : 200), Nrf2 (Abcam, Cambridge, UK, 1 : 250), HO-1 (Stressgen, Victoria, BC, Canada, 1 : 2000), copper zinc superoxide dismutase (CuZnSOD) (Santa Cruz Biotechnology, 1 : 500), manganese superoxide dismutase (MnSOD) (Stressgen, 1 : 5000), and *α*-tubulin (Sigma Chemical Co., St. Louis, MO, USA, 1 : 4000), washed, and incubated with horseradish peroxidase-conjugated antibody (Santa Cruz Biotechnology). Detection was performed using the ECL chemiluminescence (Santa Cruz Biotechnology) according to the manufacturer's instructions. The luminescent signal was recorded and quantified with the Syngene G box (Syngene, Cambridge,UK).

### 2.8. Statistical Analysis

Results are expressed as the mean ± S.E.M. Statistical analysis of differences between mean values was performed using the one-way ANOVA, followed by the Duncan's multiple range test. Differences were defined as significant at *P* < 0.05. 

## 3. Results

### 3.1. Effect of HFCR on Body Weight Change and Food Intake

**Figure 1:**
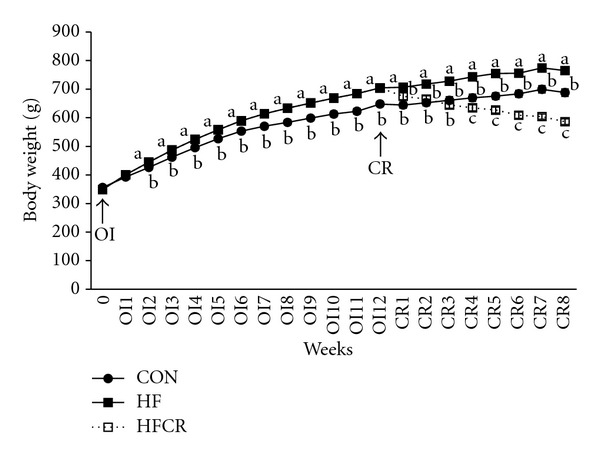
Effect of HFCR on body weight change in high-fat diet induced obese rats. OI: obesity induction, CR: calorie restriction, CON: rats fed a control diet (10% kcal fat) *ad libitum* for the experimental periods, HF: rats fed a high-fat diet (45% kcal fat) *ad libitum* for the experimental periods, HFCR: 40% calorie restricted rats fed a high-fat diet for 8–10 weeks after being fed the high-fat diet *ad libitum* for 11–13 weeks. Values are means ± SEM, *n* = 20 in each. Means for a variable without a common letter differ, *P* < 0.05.

Male SD rats were maintained on the control or the high-fat diets for 11–13 weeks. There was no significant difference in the average weight of the rats at the beginning of the study. Rats gradually developed obesity when placed on high-fat diets. After 2 weeks, the rats on the high-fat diet had significantly higher body weights than the control group. After 11–13 weeks of high-fat feeding, the HF group gained 19.74% more mass than the CON group (Figure 1). Once obesity was reached by the animals on a high-fat diet the HF group was put on a calorie restriction diet (HFCR). The CR diet was 60% of the food intake amount of the HF group. HFCR treatment significantly decreased body weights throughout the experimental periods. At the end of the 8 weeks, HFCR animals lost 118.0 g. As shown in Table 1, the food intake was significantly higher in the CON than in the HF during obesity induction period. However, energy intake was higher in the HF than in the CON. During HFCR treatment period, the HFCR showed significantly lower food and energy intake than the HF.

### 3.2. Effect of HFCR on Body Fat Pad Weight

**Figure 2:**
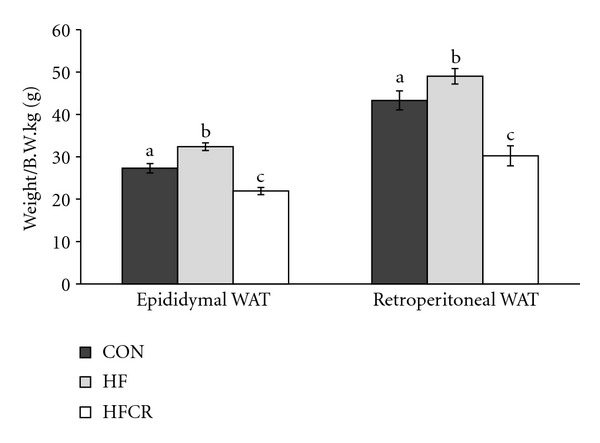
Effect of HFCR on body fat mass in high-fat diet-induced obese rats. WAT: white adipose tissue, CON: rats fed a control diet (10% kcal fat) *ad libitum* for the experimental periods, HF: rats fed a high-fat diet (45% kcal fat) *ad libitum* for the experimental periods, HFCR: 40% calorie restricted rats fed a high-fat diet for 8–10 weeks after being fed the high-fat diet *ad libitum* for 11–13 weeks. Values are means ± SEM, *n* = 20 in each. Means for a variable without a common letter differ, *P* < 0.05.

As expected, epididymal and retroperitoneal WAT were elevated in the HF when compared to the CON. HFCR for 8–10 weeks reduced epididymal WAT by 32.3% in the HF and retroperitoneal WAT by 38.3% in HF (Figure 2).

### 3.3. Effect of HFCR on Glucose Tolerance

**Figure 3:**
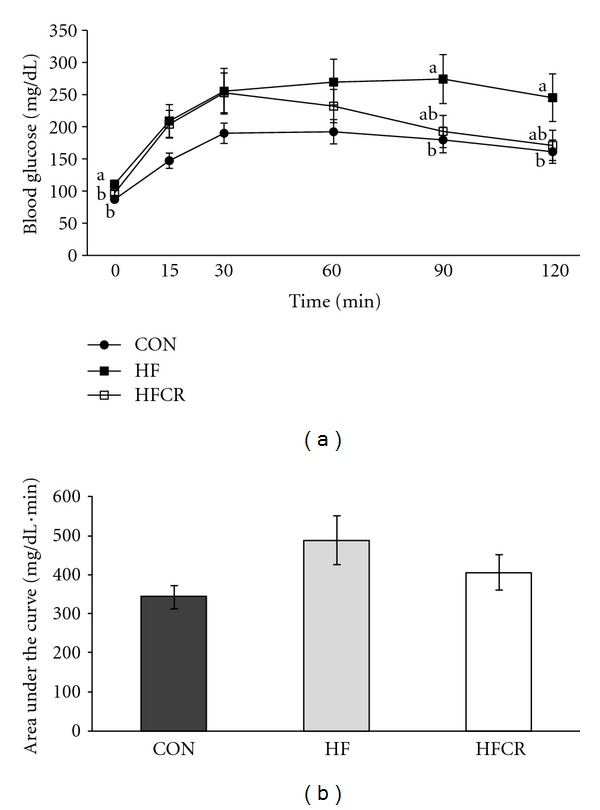
Effect of HFCR on glucose tolerance (a) and longitudinal analysis of AUC (b) in high-fat diet induced obese rats. CON: rats fed a control diet (10% kcal fat) *ad libitum* for the experimental periods, HF: rats fed a high-fat diet (45% kcal fat) *ad libitum* for the experimental periods, HFCR: 40% calorie restricted rats fed a high-fat diet for 8–10 weeks after being fed the high-fat diet *ad libitum* for 11–13 weeks, Values are means ± SEM, *n* = 20 in each. Means for a variable without a common letter differ, *P* < 0.05.

IPGTT was performed to evaluate insulin sensitivity. As shown in Figure 3(a), the HF group showed an impaired glucose tolerance. Compared to the HF, glucose tolerance curves in the HFCR were steeper after 30 minutes of glucose administration. Although there was no significant difference in the AUC of the IPGTT among the groups, there was a general trend of increased AUC of the IPGTT in the HF when compared with the CON (CON = 343.05 ± 30.27 mg/dL·min; HF = 488.60 ± 63.33 mg/dL·min; *P* = .053) (Figure 3(b)). Finally, even if it was not statistically significant, HFCR treatment decreased the obesity-associated glucose intolerance.

### 3.4. Effect of HFCR on Change of Liver and Epididymal WAT Morphology

**Figure 4:**
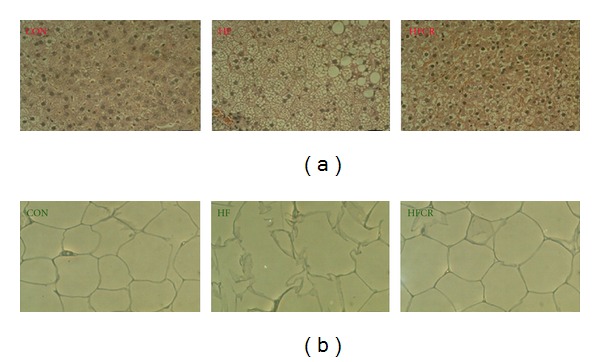
Effect of HFCR on liver and epididymal WAT morphology in high-fat diet induced obese rats. (a) H and E staining of liver from the three groups of animals (magnification X 40). The scale bar represents 250 *μ*m. (b) representative H and E staining of adipose depots from the three groups of animals (magnification X 20). The scale bar represents 250 *μ*m. CON: rats fed a control diet (10% kcal fat) *ad libitum* for the experimental periods, HF: rats fed a high-fat diet *ad libitum* for the experimental periods, and HFCR: 40% calorie-restricted rats fed a high-fat diet (45% kcal fat) for 8–10 weeks after being fed the high-fat diet *ad libitum* for 11–13 weeks.

Photomicrographs of liver and epididymal WAT sections stained with H and E are shown in Figure 4. The morphological changes (fatty liver-like) observed in the HF were reversed in the HFCR (Figure 4(a)). In particular, the HFCR did not show the extensive hypertrophy or the very large unilocular adipocytes lipid droplets that are present in the HF (Figure 4(b)).

### 3.5. Effect of HFCR on Liver Lipid Peroxidation Levels

**Figure 5:**
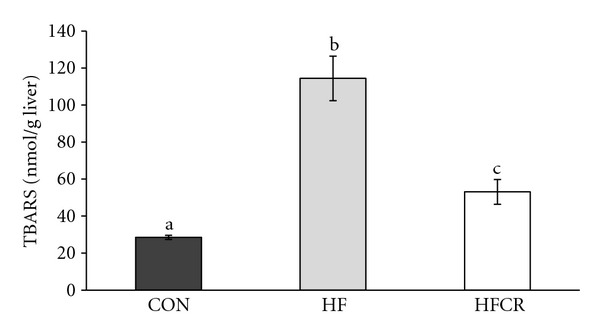
Effect of HFCR on lipid peroxidation in liver of high-fat diet induced obese rats. CON: rats fed a control diet (10% kcal fat) *ad libitum* for the experimental periods, HF: rats fed a high-fat diet *ad libitum* for the experimental periods, and HFCR: 40% calorie-restricted rats fed a high-fat diet (45% kcal fat) for 8–10 weeks after being fed the high-fat diet *ad libitum* for 11–13 weeks. Values are means ± SEM, *n* = 20 in each. Means for a variable without a common letter differ, *P* < 0.05.

As shown in Figure 5, the HF displayed a significant increase of lipid peroxidation compared to the CON (4 fold), while the HFCR displayed levels of lipid peroxidation close to the control group levels. A reduction of this magnitude was not present in the HF.

### 3.6. Effect of HFCR on Hepatic Lipids Levels

**Figure 6:**
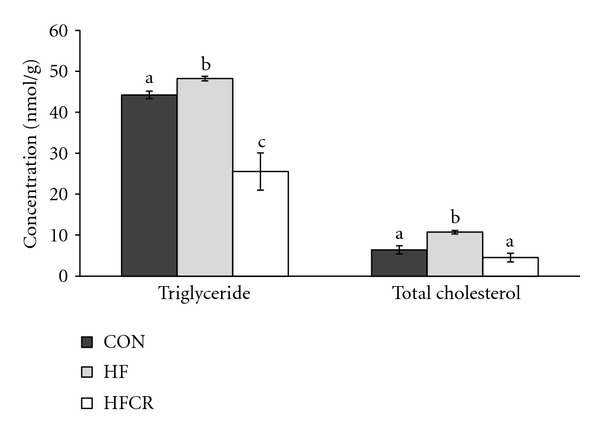
Effect of HFCR on liver lipid levels in high-fat diet-induced obese rats. CON: rats fed a control diet (10% kcal fat) *ad libitum* for the experimental periods, HF: rats fed a high-fat diet *ad libitum* for the experimental periods, HFCR: 40% calorie-restricted rats fed a high-fat diet (45% kcal fat) for 8–10 weeks after being fed the high-fat diet *ad libitum* for 11–13 weeks. Values are means ± SEM, *n* = 20 in each. Means for a variable without a common letter differ, *P* < 0.05.

The content of triglyceride and total cholesterol in the liver was increased in the HF compared with the CON. The HFCR treatment significantly reduced the presence of hepatic lipids compared to the HF (Figure 6).

### 3.7. Effect of HFCR on Plasma Adipokine Levels

**Figure 7:**
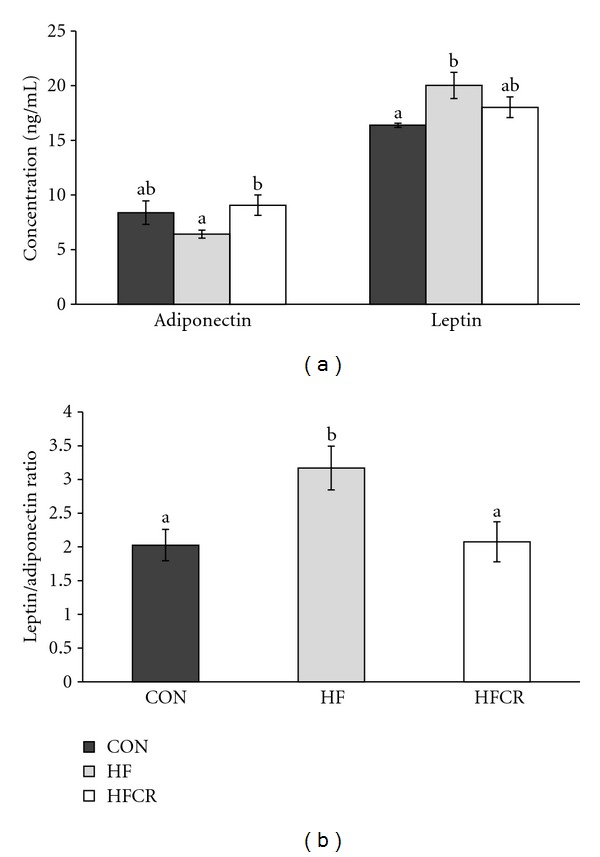
Effect of HFCR on adiponectin and leptin levels (a) and leptin/adiponectin ratio (b) in plasma of high-fat diet induced obese rats. CON: rats fed a control diet (10% kcal fat) *ad libitum* for the experimental periods, HF: rats fed a high-fat diet *ad libitum* for the experimental periods, HFCR: 40% calorie restricted rats fed a high-fat diet (45% kcal fat) for 8–10 weeks after being fed the high-fat diet *ad libitum* for 11–13 weeks, Values are means ± SEM, *n* = 20 in each. Means for a variable without a common letter differ, *P* < 0.05.

As shown in Figure 7, there was a decrease (although not significant) in adiponectin levels in the HF as compared with the CON (Figure 7(a)), this effect was completely reversed in the HFCR. The same results were observed for leptin, with a significant increase in the HF levels and a similar value between the CON and the HFCR. As a useful inflammatory biomarker, we calculate the leptin/adiponectin ratio. As shown in Figure 7(b), this ratio clearly increased in the HF when compared to the CON, while the HFCR treatment significantly lowered the ratio (Figure 7(b)).

### 3.8. Effect of HFCR on Liver Protein Expression Related to Inflammation and Oxidative Stress

**Figure 8:**
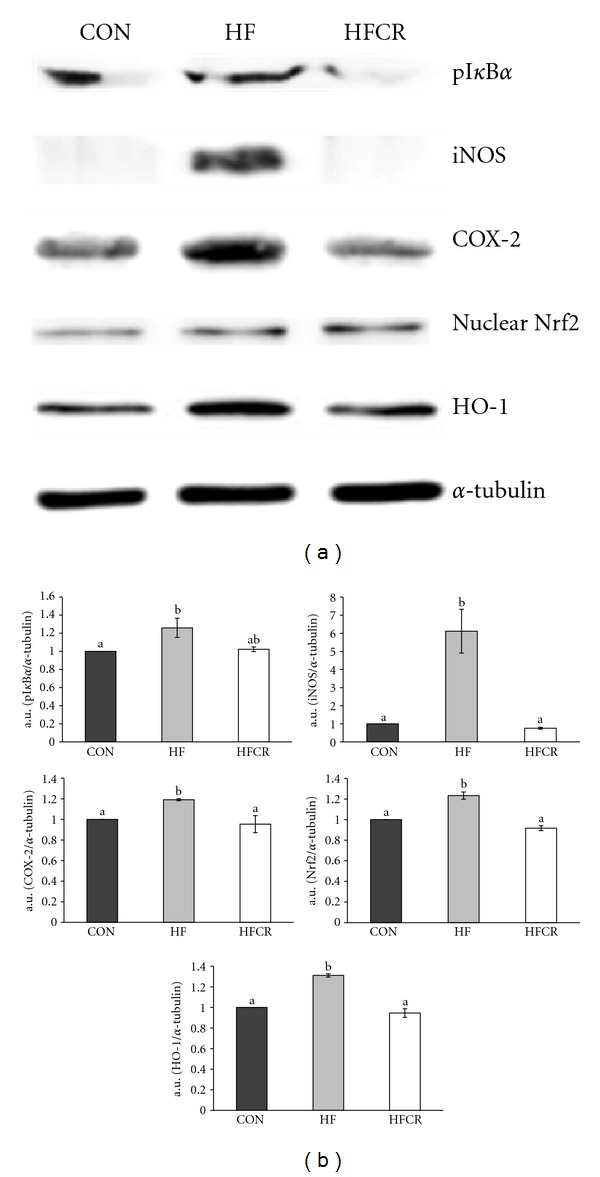
Effect of HFCR on protein expression related to inflammation and oxidative stress in liver. (a) Western blot assay of pI*κ*B*α*, iNOS, COX-2, Nrf2, and HO-1. (b) densitometric analysis of bands in Western blot. CON: rats fed a control diet (10% kcal fat) *ad libitum* for the experimental periods, HF: rats fed a high-fat diet (45% kcal fat) *ad libitum* for the experimental periods, and HFCR: 40% calorie restricted rats fed a high-fat diet for 8–10 weeks after being fed the high-fat diet *ad libitum* for 11–13 weeks. a.u.: arbitrary unit. Values are means ± SEM, *n* = 10 in each. Means for a variable without a common letter differ, *P* < 0.05.

In this study, we performed Western blot analysis to determine liver protein levels for inflammatory markers such as pI*κ*B*α* (indirect approach to evaluate NF*κ*B activation), iNOS, COX-2, and CRP. Our results showed a significant increase in pI*κ*B*α* levels in the HF, when compared with the CON levels (Figure 8). The HFCR pI*κ*B*α* levels were similar to the CON pI*κ*B*α* levels. In addition, iNOS protein expression significantly increased in the HF, and this effect was completely abolished in the HFCR that was compared to the CON. As shown in Figure 8, the high-fat diet induced COX-2 expression (*P* < 0.05 versus CON) in the HF was almost completely restored to normal levels in the HFCR. There was no significant difference with the CRP protein expression in all groups (data not shown). The HF diet caused the increase of nuclear Nrf2 protein in the HF, and the upregulation of Nrf2 was restored in HFCR treatment. The protein expression of HO-1 was increased significantly in the HF but not in the HFCR. There were no significant differences in the CuZnSOD and MnSOD protein expression in liver (data not shown).

### 3.9. Effect of HFCR on Protein Expression Related to Inflammation and Oxidative Stress in Epididymal Adipose Tissue

**Figure 9:**
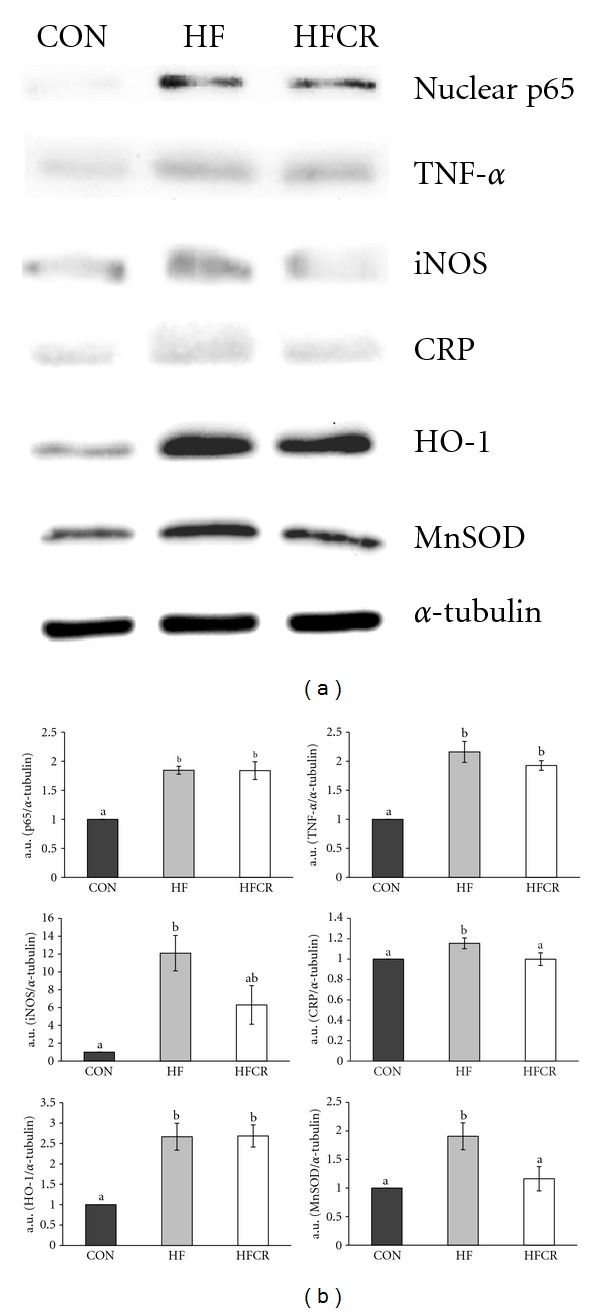
Effect of HFCR on protein expression related to inflammation and oxidative stress in epididymal adipose tissue. (a) Western blot assay of p65, iNOS, HO-1, TNF-*α*, CRP, and MnSOD. (b) densitometric analysis of bands in Western blot. CON: rats fed a control diet (10% kcal fat) *ad libitum* for the experimental periods, HF: rats fed a high-fat diet *ad libitum* for the experimental periods, HFCR: 40% calorie restricted rats fed a high-fat diet (45% kcal fat) for 8–10 weeks after being fed the high-fat diet *ad libitum* for 11–13 weeks. a.u.: arbitrary unit. Values are means ± SEM, *n* = 10 in each. Means for a variable without a common letter differ, *P* < 0.05.

Protein expression level related to inflammation (p65, TNF-*α*, iNOS, and CRP) and oxidative stress (HO-1, CuZnSOD, and MnSOD) from adipose tissue in each treatment group was determined by Western blot. The nuclear p65 and TNF-*α* expression levels were significantly higher in the HF than in the CON (Figure 9). The HFCR did not induce this increase. In the HF animals, the level of iNOS protein expression significantly increased, compared to the CON. The HFCR lowered significantly. The same trend was observed for CRP, an acute-phase protein that rises in response to inflammation. As shown in Figure 9, induction of HO-1 was higher in the HF than in the CON. No effect was noticed with the induction of HO-1 in the HFCR. The protein expression levels of MnSOD in epididymal WAT increased in the HF group, with respect to the CON. The COX-2 expression levels were back to steady levels in the HFCR. The protein expression of CuZnSOD in epididymal WAT did not differ among the groups (data not shown).

## 4. Discussion

The present study was performed in order to evaluate the possible effect of HFCR on the induction of inflammation and oxidative stress damage by a high-fat diet.

We demonstrated HFCR-reduced metabolic abnormalities, such as dyslipidemia and plasma adipokine levels, in the obese rats continuously fed the HF diet. The HFCR animals gained less body weight, accumulated lower body fat, hepatic triglyceride, and cholesterol than HF animals, which is a similar pattern of previous studies [21, 28]. HFCR decreased the levels of lipid peroxidation demonstrated by MDA, a generally accepted biomarkers of lipid peroxidation [29]. Our data suggests that HFCR as well as CR maintains the redox-balancing power in cells and tissues [19, 30] and exhibits reduced oxidative damage. Our data showed that HFCR tends to reduce basal fasting blood glucose and impairment of glucose tolerance in the HF, which is in parallel with previous reports [21, 22]. Barzilai and Gabriely suggested the beneficial effect of dietary CR on glucose homeostasis can be attributed to a decrease in adipose cells and their products [31]. In addition, steatosis (fatty liver) shown in the HF disappeared in the HFCR and extensive hypertrophy of the WAT in obese rats was reversed by HFCR treatment as compared to that in the CON.

Adipose tissue is no longer considered to be solely an energy-storage tissue [32]. Adipose tissue produces and secretes a variety of signals and factors associated with inflammation, including IL-6, IL-1*β*, TNF-*α*, MCP-1, and adipokines such as adiponectin, leptin, and resistin [4, 5]. Adiponectin is an essential mediator in the regulation of insulin resistance and antiinflammatory effects through the inhibition of TNF-*α* and upregulation of the antiinflammatory cytokines [32–34]. In our study, the levels of plasma adiponectin were decreased in the HF, while HFCR treatment increased the levels of plasma adiponectin. Leptin, an adipocyte-derived hormone, regulates energy intake and energy expenditure [35]. In obese humans and rodents, leptin resistance, which is circulating leptin, fails to reach its targets in the brain. [36, 37]. Therefore, obesity leads to an increase in the circulating leptin levels. Recent studies have reported the leptin/adiponectin ratio is correlated with body mass index and may be a useful biomarker for inflammation, insulin resistance, and atherogenesis [38–41]. In the present study, an increased ratio of leptin/adiponectin of the HF group was significantly decreased by the HFCR treatment.

Consumption of a high-fat diet has been reported to promote inflammation and the activation of NF*κ*B [42]. In a normal state, NF*κ*B is bound with an inhibitory protein of nuclear factor-*κ*B (I*κ*B) in the cytoplasm. Following stimuli, such as oxidative stress and various cytokines, I*κ*B is phosphorylated and NF*κ*B is free to migrate into the nuclei and activate several genes such as iNOS and COX-2 [43–45]. Our results have shown that intake of a high-fat diet activated NF*κ*B p65 subunit in rat liver. Previous research reported NF*κ*B binding activity was higher in the rats fed high-fat diets than rats fed control diets [46].

The inflammation response of iNOS may be an important factor because it can be induced by several inflammatory stimuli [47]. It has been shown that iNOS expression in the liver has been shown to increase inflammation in the ob/ob mice. Treatment with an iNOS inhibitor reversed fasting hyperglycemia. iNOS inhibitors improved insulin sensitivity by increasing the protein expression of IRSs and enhancing IRSs-mediated insulin signaling in the liver of ob/ob mice [48]. Our study showed liver iNOS expression pattern in the HF group was similar to the previous study where the increase of iNOS expression in ob/ob mice [48].

Cumulative evidence shows that COX-2 activation contributes to the generation of ROS in the pathophysiological condition and mediates the Nrf2 activation by regulating inflammatory response and transcriptional activity [49]. Our data showed the HFCR treatment, which decreased the COX-2 protein level, not only attenuated obesity-related increase in liver oxidative damage, but indicated a suppression of elevated liver contents of TBARS, an index of oxidative damage. The HFCR treatment suppressed nuclear Nrf2 and HO-1 protein expression in liver. Helmersson et al. [50] demonstrated that the development of type 2 diabetes mellitus (T2DM) in elderly men was significantly correlated with COX-mediated inflammation and oxidative stress. The beneficial effects of CR can be further extended to its ability to modulate proinflammatory proteins, such as IL-1*β*, IL-6, TNF-*α*, and iNOS as well as COX-2 mRNA and protein level by manipulating NF*κ*B [51]. A recent study identified Nrf2 as a pivotal transcription factor for controlling hepatic oxidative stress [52]. The induction of Nrf2 activity though CR is known to decrease ROS production, decreases inflammation processes, and improves insulin signaling pathways [53].

Nrf2 regulates the antioxidant response element (ARE)-dependent gene regulation in the response to oxidative stress. Nrf2 induces expression of antioxidant enzymes such as HO-1 by binding to ARE in the promoters of these genes [54]. Our data clearly shows the HF treatment led to an increase of Nrf2 in liver, along with raised levels of HO-1, whereas the effect was reversed by the HFCR treatment. Based on this information, we suggest that ROS overproduction in obesity can activate Nrf2.

Adipose tissue is an important source of TNF-*α*, a major proinflammatory factor in obesity [55]. TNF-*α* contributes insulin resistance by blunting the insulin-stimulated tyrosine phosphorylation of IRS-1 [56], inhibiting glucose uptake [57], and activating NF*κ*B pathway [58]. Our data showed in the HF groups that there was an increase of adipose tissue protein levels of TNF-*α*. Although the HFCR treatment did not significantly decrease TNF-*α* protein levels in adipose tissues, HFCR treatment resulted in the improvement of glucose intolerance. These results suggest that other inflammatory factors may indirectly correlate with insulin sensitivity. Moreover an upregulation of TNF-*α* mediates an increase in NF*κ*B nuclear translocation, which results in the activation of inflammatory genes expression such as iNOS and CRP [59, 60]. In this study, the HFCR treatment was not able to reduce the nuclear p65 expression in epidydimal WAT but significantly reduced a downstream gene, iNOS, and an inflammation related protein, CRP, in epididymal WAT as compared with the HF. Previous studies reported CR's antiinflammatory action such as suppression of NO production by inhibition of iNOS in alveolar macrophage [61] and reduction of plasma CRP levels, indicating a reduction of systemic inflammation [62]. The proper levels of ROS during inflammation serve as essential regulators in the signal transduction pathway [63–65]. HFCR treatment decreased expression of important inducible antioxidant enzymes, MnSOD, along with reduced oxidative damage was demonstrated by lipid peroxidation in this study. These results suggest HFCR still has beneficial antioxidant and antiinflammatory capacity.

## 5. Conclusion

Collectively, the present results show that HFCR without a dietary composition change decreased the leptin/adiponectin ratio in plasma, suppressed the proinflammatory cytokines expression in epidydimal WAT, and reduced the inflammation and oxidative damage in the liver and epidydimal WAT. In conclusion, HFCR and CR with a low-fat diet are still beneficial approaches to obese subjects. These diets increase long-term compliance in weight control which induce attenuation of obesity-induced inflammatory responses, which is a risk factor for obesity related chronic diseases, along with decreased fat mass in obesity.

## Figures and Tables

**Table 1:** Daily food and energy intakes in the experimental animals.

Group	Before HFCR		After HFCR	
	Food intake (g/day)	Energy intake (kcal/day)	Food intake (g/day)	Energy intake (kcal/day)
CON	25.92 ± 0.13^a^	99.79 ± 0.50^a^	24.72 ± 0.23^a^	95.16 ± 0.87^a^
HF	23.37 ± 0.13^b^	110.53 ± 0.64^b^	21.67 ± 0.20^b^	102.50 ± 0.94^b^
HFCR			13.01 ± 0.04^c^	61.56 ± 0.21^c^

CON: rats fed a control diet (10% kcal fat) *ad libitum* for the experimental periods, HF: rats fed a high-fat diet (45% kcal fat) *ad libitum* for the experimental periods, HFCR: 40% calorie restricted rats fed a high-fat diet for 8–10 weeks after being fed the high-fat diet *ad libitum* for 11–13 weeks. Values are means ± SEM, *n* = 20 in each group. Means for a variable without a common letter differ, *P* < 0.05.

## Abbreviations

CR: Calorie restriction
IL: Interleukin
TNF: Tumor necrosis factor
MCP: Monocyte chemoattractant protein
JNK: Jun N-terminal kinase
IKK: I*κ*B kinase
NF: Nuclear factor
IRS: Insulin receptor substrate
PI3: Phosphatidylinositol 3
GLUT: Glucose transporter
CRP: C-reactive protein
COX: Cyclooxygenase
iNOS: Inducible nitric oxide synthase
Nrf2: NF-E2-related factor 2
HO: Heme oxygenase
HF: High-fat
SD: Sprague-Dawley
CON: A control diet group
HF: A high-fat diet group
HFCR: The high-fat diet group with calorie restriction
WAT: White adipose tissue
IPGTT: Intraperitoneal glucose tolerance test
i.p.: Intraperitoneally
AUC: Area under the glucose curve
H and E: Hematoxylin and eosin
MDA-TBA: Malondialdehyde-thiobarbituric acid
PVDF: Polyvinylidene fluoride
CuZnSOD: Copper zinc superoxide dismutase
MnSOD: Manganese superoxide dismutase
I*κ*B: Inhibitory protein of nuclear factor-*κ*B
T2DM: Type 2 diabetes mellitus
ARE: Antioxidant response element
a.u.: Arbitrary unit.
